# Supplementary material for: Generalized cue reactivity in rat dopamine neurons after opioids
Source: Nat Commun. 2025 Jan 2;16:321. doi: 10.1038/s41467-024-55504-3 (PMC11697388; doi:10.1038/s41467-024-55504-3)
Supplement: Supplementary file 1 — Supplementary Information [file 41467_2024_55504_MOESM1_ESM.pdf]

Supplemetary Information

to Generalized cue reactivity in rat dopamine neurons after opioids

Authors: Collin M. Lehmann, Nora E. Miller, Varun S. Nair, Kauê M. Costa, Geoffrey Schoenbaum, Khaled Moussawi

Supplementary figures:

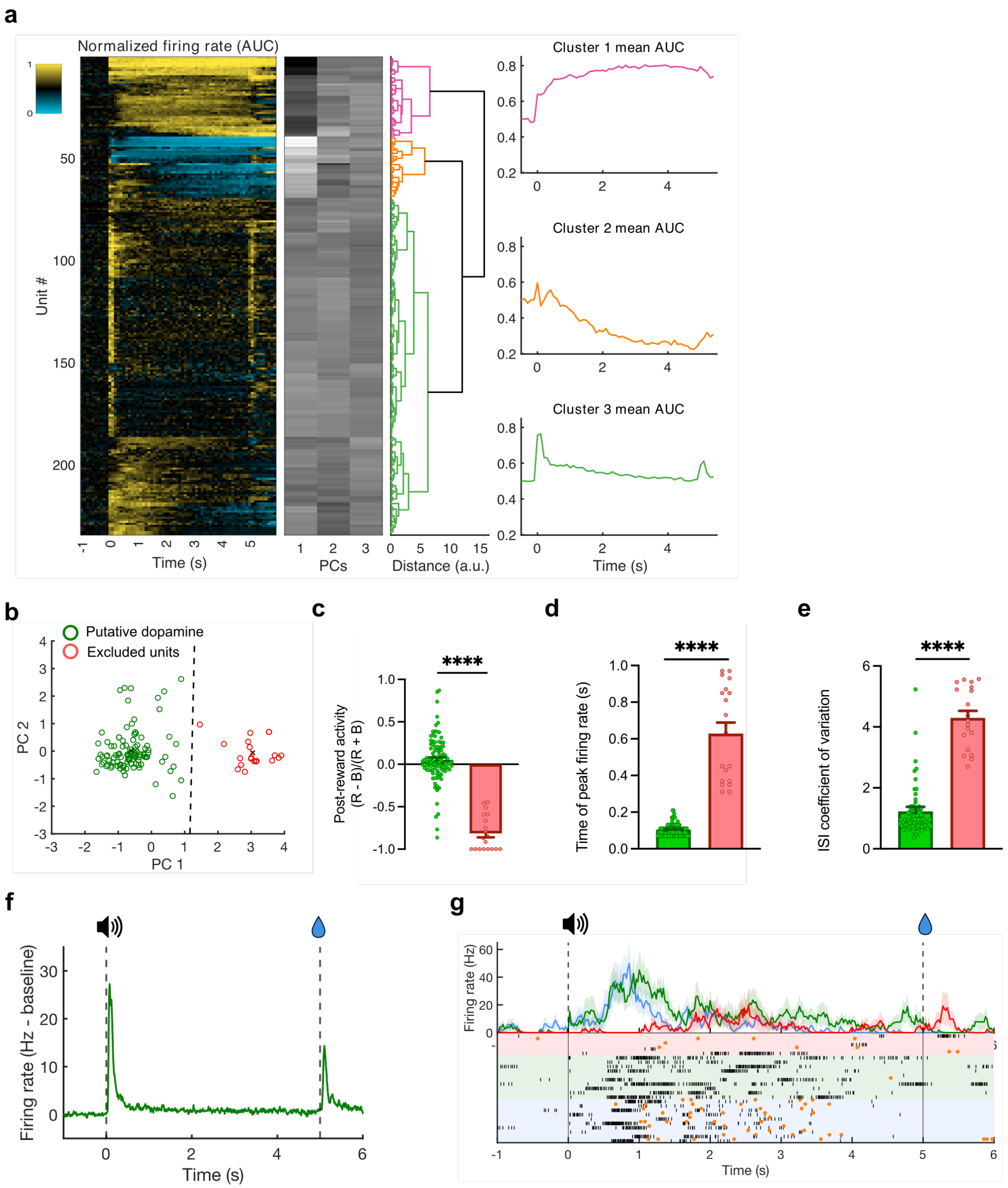

Supplementary Fig. 1. Putative dopamine neurons identification in Experiment 1.

(**a**) Raw unit clustering results from Experiment 1 ($N = 11$ rats). Left: heatmap showing functional activation of each unit (rows) aligned to sucrose cue onset (0 s). Activity normalized using area under receiver-operator curve (auROC) method, compared against baseline activity during 5 s before cue onset. Scales from 0 (signal perfectly discriminable, less than baseline) to 1

(perfectly discriminable, greater than baseline). Center left: first three components extracted via principal component analysis. Center: dendrogram showing results from hierarchical agglomerative clustering. Right: mean AUC normalized activity for units classified into each cluster. (**b**) Scatter plot of first two principal components extracted from peak firing 50-1000 ms after cue, peri-reward inhibition, and ISI coefficient of variation for all units in cluster 3 with baseline firing rate <= 12Hz. Green circles putative dopamine units retained by *k*-means clustering; red circles were eliminated units, with centroids marked by corresponding colored crosses. Black dashed line indicates decision boundary. (**c**) Time of peak firing 50-1000 ms after cue for retained (green) and excluded units (red); two-tailed Mann-Whitney U-test, $n = 119$, $U = 67.50$, $p < 0.0001$. (**d**) Peri-reward inhibition ($[R\text{-}B]/[R+B]$ where R = firing rate 0-1 s after reward, and B = baseline firing rate) for retained and excluded units (two-tailed Mann-Whitney U-test, $n = 119$, $U = 145$, $p < 0.0001$). (**e**) Coefficient of variation of ISIs for spikes recorded outside phasic firing periods (~500 ms after programmed events) for retained and excluded units (two-tailed Mann-Whitney U-test, $n = 119$, $U = 21$, $p < 0.0001$). (**f**) Mean ± SEM firing rate (Hz – baseline) of units from raw phasic firing cluster that were retained as putative dopamine ($n = 100$). (**g**) Example PSTH and raster session data from one eliminated neuron from the original cluster 3. Vertical lines indicate cue onset (0 s) and cue offset/sucrose delivery (5 s). Trials binned at 20 ms for clarity. All traces (red = neutral trials, green = remifentanil, and blue = sucrose) and shaded outlines indicate mean ± SEM. Raster plot shows individual trials. Black ticks mark recorded spikes. Orange dots indicate sucrose port entries. Source data are provided as a Source Data file.

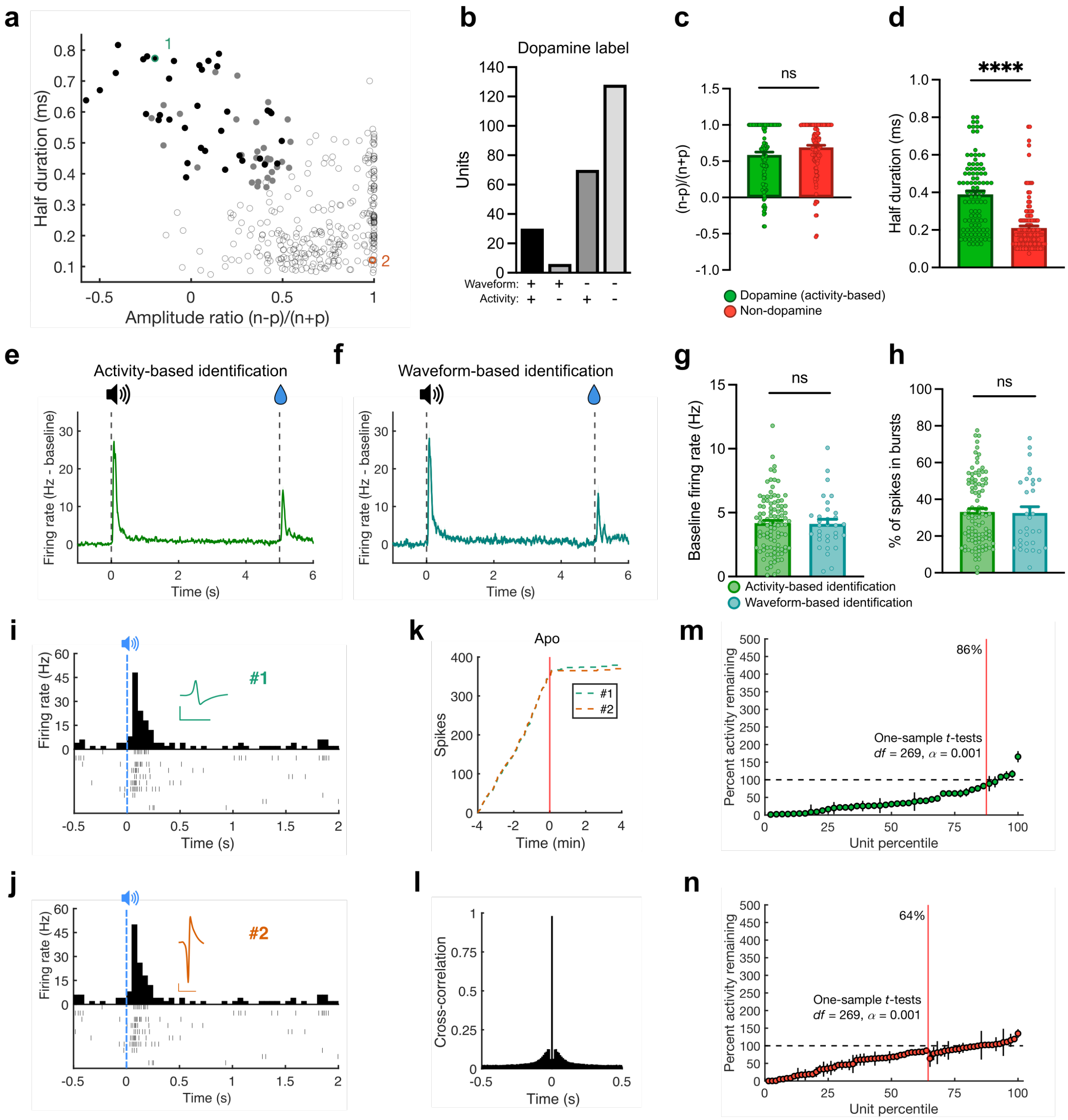

Supplementary Fig. 2. Waveform-based identification of dopamine neurons was more conservative but consistent with functional clustering.

(**a**) Scatter plot of all units by half-duration and amplitude ratio ($N = 11$ rats). Black-filled circles are identified as dopamine neurons by waveform-based *k*-means clustering [71,75]. Gray-filled circles are non-responsive units in the activity-based dopamine cluster from Fig. 2. Empty, semi-opaque circles are classified as non-dopamine. Identified units 1 and 2 were recorded in different

channels in the same session and likely represent the same neuron; this is described further in panels e-h. (**b**) Number of units identified as dopamine by waveform criteria (panel a) and activity-based criteria in Fig. 2. (**c**) Amplitude ratios of units identified as dopamine by activity-based method (two-tailed Mann-Whitney U-test, $n = 234$, $U = 5920$, $p = 0.1304$). (**d**) Half-durations of units identified as dopamine by activity-based method (two-tailed Mann-Whitney U-test, $n = 234$, $U = 2791$, $p < 0.0001$). (**e**) Trace of mean ± SEM firing response during sucrose trials for units identified by activity-based clustering ($n = 99$). (**f**) Trace of mean ± SEM firing response during sucrose trials for units identified by waveform criteria ($n = 32$). (**g**) Baseline firing rates for neurons identified as dopamine based on sucrose trial spiking activity (green) and waveform properties (teal; two-tailed Mann-Whitney U test, $n = 131$, $U = 1531$, $p = 0.7765$). (**h**) Percent of recorded spikes occurring in bursts for neurons identified as dopamine based on sucrose trial spiking activity and waveform properties (two-tailed Mann-Whitney U test, $n = 131$, $U = 1536$, $p = 0.7970$). (**i**) Raster plots and PSTH of example responses to sucrose cue from Unit 1 in panel A, identified as dopaminergic by waveform properties. Waveform inset at scale of 500 ms, 75 μV. (**j**) Raster plots and PSTH from Unit 2 in panel A identified as non-dopaminergic, during the same behavioral trials as in panel I (both units recorded in different channels during the same session). Note that firing of the 2 units appears identical in response to the sucrose cue. (**k**) Cumulative spikes around IV infusion of 20 μg/kg apomorphine for units 1 and 2. (**l**) Cross-correlation of spikes in units 1 and 2. Note correlation is approximately 1 at zero delay suggesting the two recordings represent the same neuron. (**m**) 86% of dopamine neurons show inhibition of firing after apomorphine infusion. Neuronal firing relative to baseline (mean ± 95% CI) after IV apomorphine infusion in activity-identified dopamine neurons (baseline: five min prior to infusion; mean activity: 5 min after infusion). For each individual neuron, firing in the interval 30-300 s post-infusion was divided into 1 s intervals, and tested with a one-sample *t*-test

for difference from pre-infusion mean baseline firing rate at $\alpha = 0.001$. Vertical bars indicate 95% confidence interval of inhibition, with large bars suggesting greater variability of post-infusion firing rate. Horizontal black dashed line indicates line of no-effect. Vertical red line separates units with significant from non-significant response inhibition by apomorphine. (**n**) Same as panel m but for activity-identified non-dopamine neurons. Source data are provided as a Source Data file.

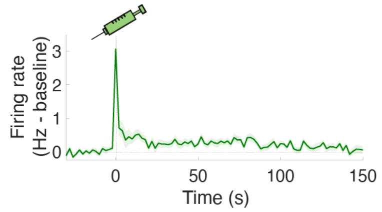

Supplementary Fig 3. Persistent dopamine neuron response to 4 μg/kg remifentanil in opioid-experienced rats

Trace (mean ± SEM) of firing responses to cued remifentanil infusion of 4 μg/kg across recordings (*n* =31 units, *N* = 7 rats) after months of opioid exposure. Source data are provided as a Source Data file.

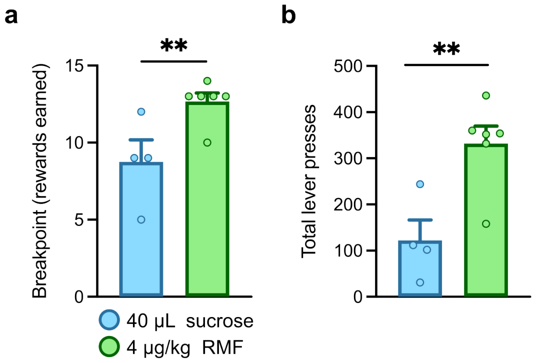

Supplementary Fig 4. Breakpoint for remifentanil and sucrose in a progressive ratio schedule of reinforcement.

(**a**) Training was conducted at the end of Experiment 1 in the opioid-exposed group (N = 6 rats). Final step achieved during progressive ratio (PR) test in which lever press requirement to receive reward (40 µL sucrose or 4 µg/kg remifentanil [RMF]) was increased according to an exponential schedule [73] (steps = 1, 2, 4, 6, 9, 12, 15, 20, 25, 32, 40, 50, 62, 77, 95, 118, 145, 178, 219, 268, 328, 402, 492, 603, and 737). Each point represents the highest level achieved out of ~3 PR attempts per rat (two-tailed Mann-Whitney U-test, $n = 10$, $U = 1$, $p = 0.0095$). All bars show mean + SEM. Note that initially 6 rats were trained to respond for remifentanil. One rat failed to acquire lever pressing behavior for sucrose reward, and one rat was sacrificed before sucrose training could be completed. (**b**) Total lever presses on active lever during best PR performances for sucrose and remifentanil reward per rat (two-tailed unpaired t-test, $t(8) = 3.566$, $n = 10$, $p = 0.0073$). Source data are provided as a Source Data file.

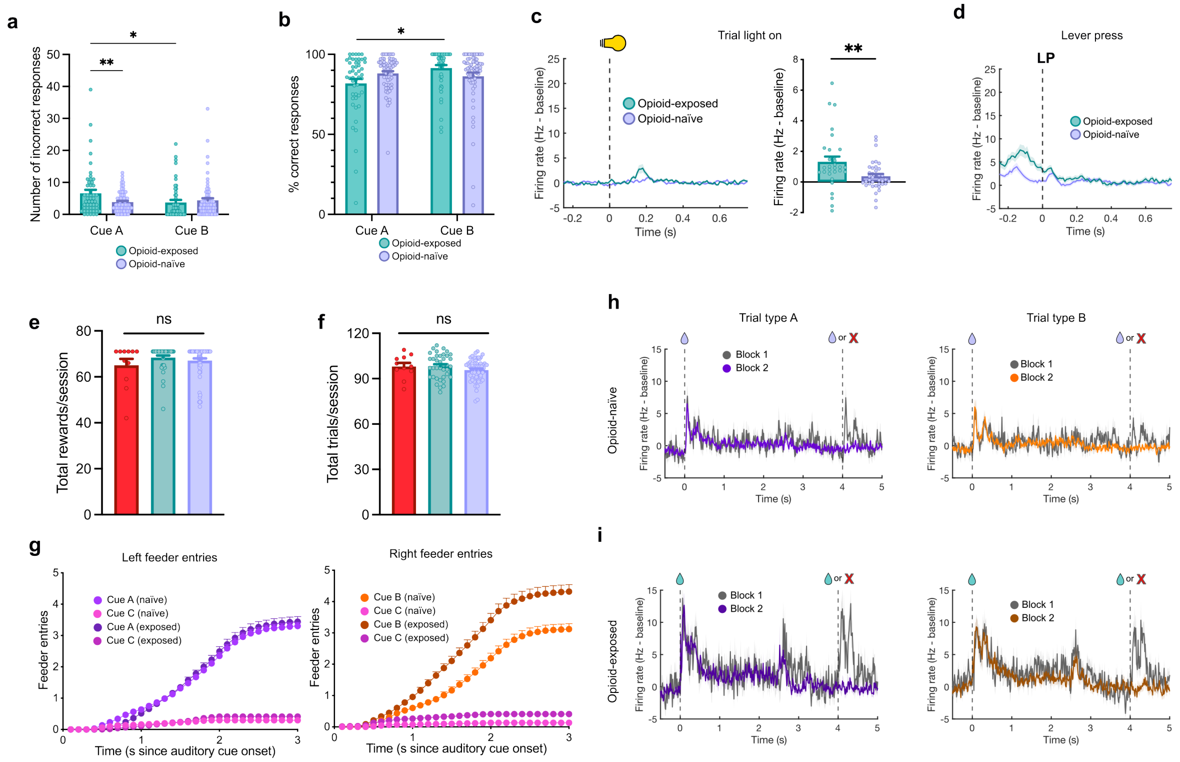

Supplementary Fig 5. Behavioral performance and dopamine firing response data from the operant procedure.

(**a**) Number of incorrect responses per recording session to Cue A and Cue B for opioid-exposed and opioid-naïve rats (N = 6 opioid-naïve rats, N = 5 opioid-exposed rats, 2-way RM ANOVA, Cue x Exposure interaction: $F(1,114) = 4.476$, $p = 0.0366$, Fisher's LSD: Cue A Exposed vs Naive, $p = 0.0089$; Cue B Exposed vs. Naive, $p = 0.5405$; Exposed Cue A vs. Cue B, $p = 0.0197$; Naive Cue A vs. Cue B, $p = 0.6152$). All bars show mean + SEM. (**b**) Percent correct feeder-in responses to auditory Cue A and Cue B per recording session in opioid-exposed and opioid-naïve rats (Cue factor: $F(1,114) = 3.245$, $p = 0.0743$; Exposure factor: $F(1,114) = 0.5252$, $p = 0.9907$; Cue x Exposure interaction $F(1,114) = 3.098$, $p = 0.0811$, Fisher's LSD: Cue A Exposed vs Naive, $p = 0.0690$; Cue B Exposed vs. Naive, $p = 0.3582$; Exposed Cue A vs. Cue B, $p = 0.0199$; Naive Cue A vs. Cue B, $p = 0.9749$). (**c**) Left: Trace of average putative dopamine responses to trial light illumination at the start of trial for opioid-exposed and opioid-naïve units, prior to lever

extension. All traces show mean ± SEM. Right: average firing response during 100-200 ms after trial light turns on (two-tailed Mann-Whitney test, n = 68, U = 330, p = 0.0027). (**d**) Trace of average putative dopamine response to lever press for opioid-exposed and opioid-naïve units. (**e**) Total number of rewards during each session for rat 8 (red) all other opioid-exposed rats (teal) and opioid-naïve rats (purple; Kruskal-Wallis test, n = 107, k = 2.328, p = 0.3122). (**f**) Total number of trials (all types) during each session for rat 8 (red) all other opioid-exposed rats (teal) and opioid-naïve rats (purple Kruskal-Wallis test, n = 107, k = 2.338, p = 0.3107). (**g**) Cumulative feeder entries at the non-drug (left panel) and drug-associated feeder (right panel) following auditory cue onset. Note low rate of responding to Cue C. (**h**) Average responses of opioid-naïve units to reward delivery in block 1 (gray) and block 2 (color), with reward one delivered at 0 s and reward two (or omission) at 4 s. Left panel shows responses to reward at left port in trial-type A (purple/gray) and right panel shows reward at right port in trial-type B (orange/gray). (**i**) Same as in G but for opioid-exposed units. Note the absence of negative reward prediction errors in either group to the second reward omission, likely because the animals are well trained and learned to expect the second reward omission. Source data are provided as a Source Data file.

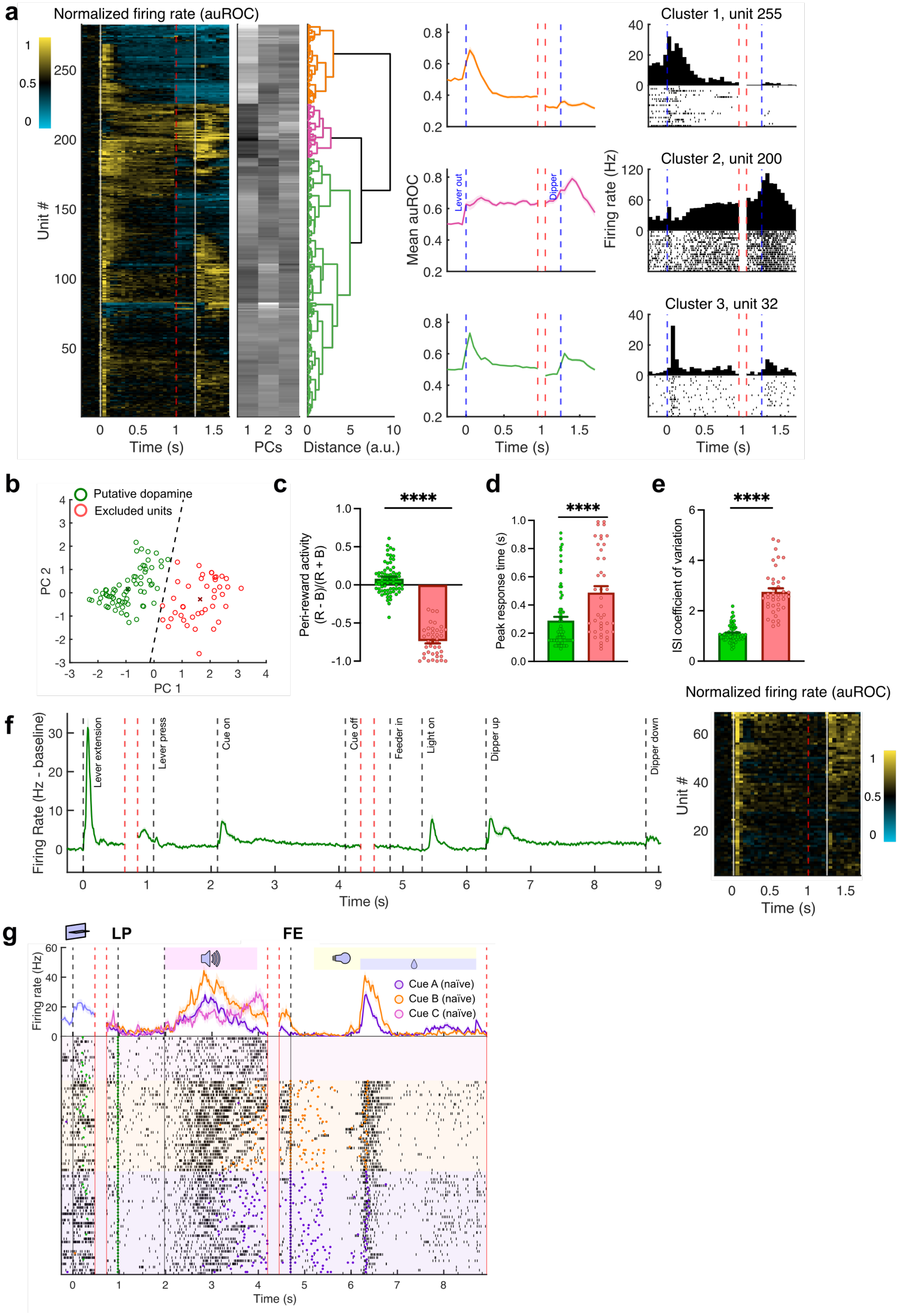

Supplementary Fig 6. Putative dopamine neurons identification in Experiment 2.

(**a**) Initial clustering results for cue-responsive units in operant experiment ($N = 11$ rats). Left: Heatmap showing functional activation of each unit (rows) centered on lever extension and water reward delivery. Events are displayed as white solid lines, and time discontinuity as dashed red line. Activity normalized using area under receiver-operator curve (auROC) method, compared against baseline. Scales from 0 to 1. Center left, first three components extracted via principal component analysis. Center: Dendrogram showing results from hierarchical clustering split at three clusters. Center right: Mean ± SEM auROC data for each group. Time discontinuity shown as white space flanked by dashed red lines. Right: example neurons from each cluster. PSTH and raster plots from example units for each cluster. (**b**) Scatter plot of first two principal components (PCs) extracted from peak firing 50-1000 ms after cue, peri-reward inhibition, and ISI coefficient of variation for all units in cluster with baseline firing rate <= 12Hz. Green circles putative dopamine units retained by k-means clustering; red circles were eliminated units, with centroids marked by corresponding colored crosses. Black dashed line indicates decision boundary. (**c**) Peri-reward inhibition ($[R\text{-}B]/[R\text{+}B]$ where R = firing rate 0-1 s either before or after reward, and B = baseline firing rate) for retained putative dopamine (green) and excluded units (red; two-tailed Mann-Whitney U-test, $n = 109$, $U = 3$, $p < 0.0001$). All bars show mean + SEM. (**d**) Time of peak firing 50-1000 ms after auditory cue for retained and excluded units (two-tailed Mann-Whitney U-test, $n = 109$, $U = 726.5$, $p < 0.0001$). (**e**) Coefficient of variation of ISIs for spikes recorded outside phasic firing periods (~500 ms after programmed events) for retained (green) and excluded units (red; two-tailed Mann-Whitney U-test, $n = 109$ $U = 45$, $p < 0.0001$). (**f**) Left; Mean ± SEM firing rate responses for neurons retained as putative dopamine neurons (n = 69). Right; corresponding auROC heatmap of final putative dopamine cluster. All traces show mean ± SEM (**g**) Example PSTH and raster session data from one eliminated neuron from the original

cluster 3. Purple and orange dots indicate left and right feeder entries respectively. Green dots indicate lever presses. Source data are provided as a Source Data file.

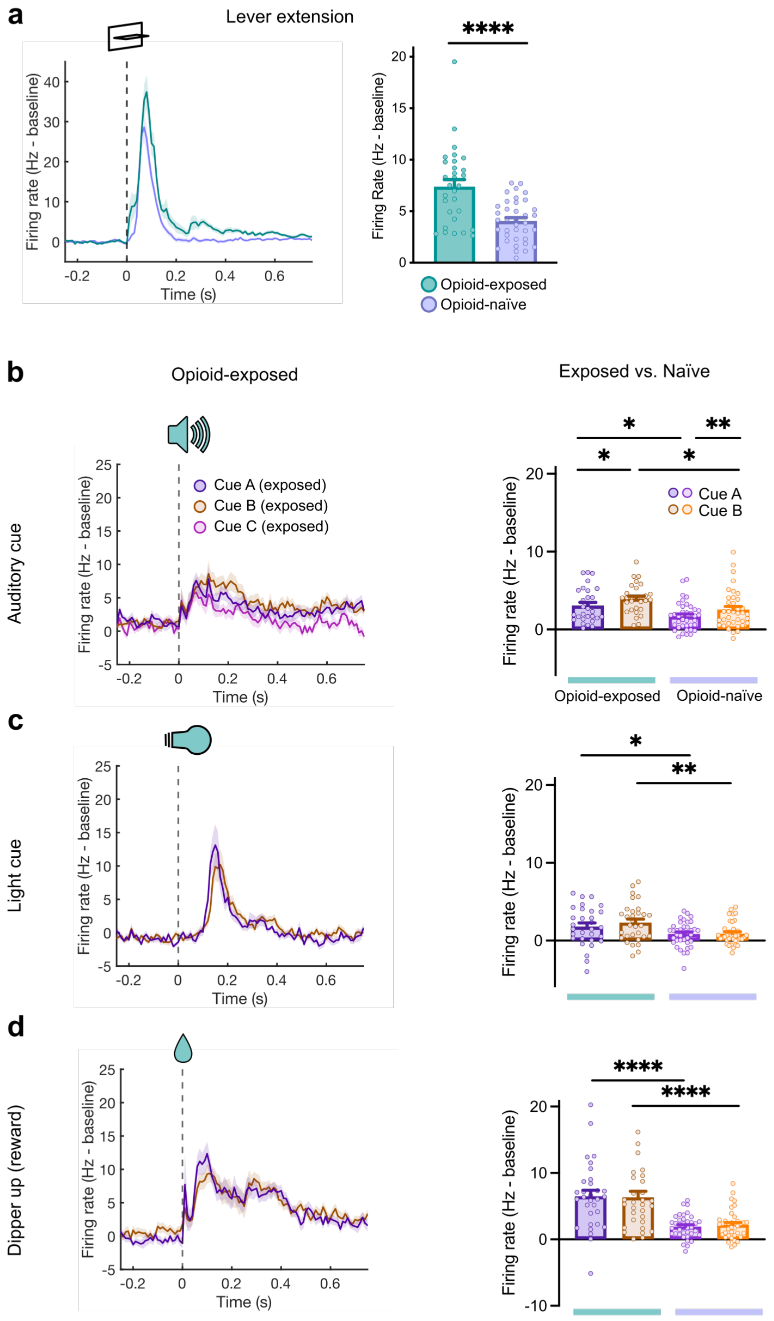

154

155 **Supplementary Fig 7. Sensitized dopamine responses to drug and non-drug cues in all**
156 **opioid-exposed rats.**

157 Dopamine firing response analyses comparing opioid-naïve ($N = 6$ rats) to opioid-exposed ($N = 5$
158 rats) units including those observed in rat 8. Panels are similar to those in Fig. 5. (**a**) Left: PSTH
159 of baseline-subtracted firing rate around lever extension for opioid-exposed and opioid-naïve

units. All traces show mean ± SEM Right: Mean firing rate for both groups (two-tailed Mann-Whitney test, $n = 67$, $U = 242$, $p < 0.0001$). All bars show mean + SEM. (**b**) PSTH of the responses for opioid-exposed units around to auditory cues. Mean firing rates for opioid-exposed and opioid-naïve units (same naïve units as in Fig. 5 g) (2-way RM mixed-effects analysis, Cue factor: $F(1,64) = 10.79$, $p = 0.0017$; Exposure factor: $F(1,65) = 8.157$, $p = 0.0058$; Cue x Exposure interaction $F(1,64) = 0.04719$, $p = 0.8287$, Fisher's LSD: Cue A Exposed vs Naive, $p = 0.0170$; Cue B Exposed vs. Naive, $p = 0.0096$; Exposed Cue A vs. Cue B, $p = 0.0226$; Naive Cue A vs. Cue B, $p = 0.0237$). (**c**) Responses of opioid-exposed and opioid-naïve neurons to light cue, similar to b. Comparison of mean baseline-subtracted firing rates light cues in both groups (Cue factor: $F(1,65) = 1.321$, $p = 0.2547$; Exposure factor: $F(1,67) = 7.537$, $p = 0.0078$; Cue x Exposure interaction $F(1,65) = 1.541$, $p = 0.2190$, Fisher's LSD: Cue A Exposed vs Naive, $p = 0.0292$; Cue B Exposed vs. Naive, $p = 0.0032$; Exposed Cue A vs. Cue B, $p = 0.1125$; Naive Cue A vs. Cue B, $p = 0.9454$). (**d**) Responses of opioid-exposed and opioid-naïve neurons to water reward delivery (dipper up). Comparison of mean baseline-subtracted firing rate after left vs. right dipper up for opioid-exposed and opioid-naïve units (Cue factor: $F(1,66) = 0.2852$, $p = 0.5951$; Exposure factor: $F(1,67) = 24.37$, $p < 0.0001$; Cue x Exposure interaction $F(1,66) = 0.003168$, $p = 0.9553$, Fisher's LSD: Cue A Exposed vs Naive, $p < 0.0001$; Cue B Exposed vs. Naive, $p < 0.0001$; Exposed Cue A vs. Cue B, $p = 0.6905$; Naive Cue A vs. Cue B, $p = 0.7245$). Source data are provided as a Source Data file.

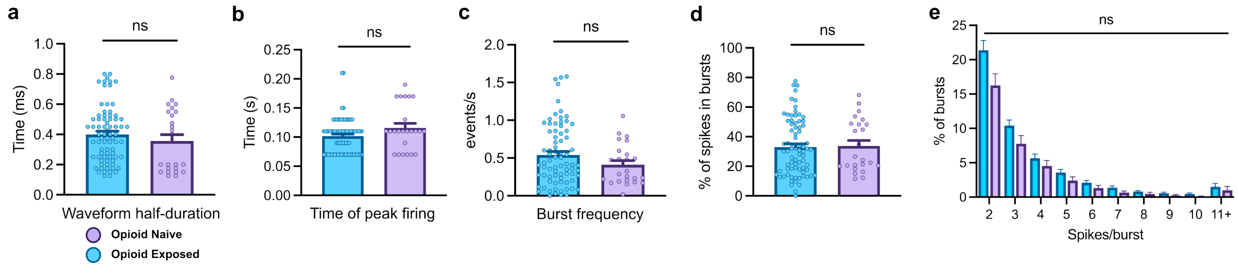

Supplementary Fig 8. Dopamine neurons waveform and firing properties between groups. (**a**) Waveform half-duration of putative dopamine neurons for opioid-exposed ($N = 7$ rats) vs. opioid-naïve ($N = 4$ rats) units from Experiment 1, defined as time from negative peak to next positive peak, or end of waveform (two-tailed Mann-Whitney test, $n = 99$, $U = 777$, $p = 0.3176$). (**b**) Time of peak firing during first second following sucrose cue onset (two-tailed Mann-Whitney test, $n = 99$, $U = 822$, $p = 0.5245$). (**c**) Frequency of bursts during intertrial interval for opioid-exposed and naïve units (two-tailed Mann-Whitney test, $n = 99$, $U = 754$, $p = 0.2357$). (**d**) Percent of all recorded intertrial interval spikes occurring in bursts (two-tailed Mann-Whitney test, $n = 99$, $U = 867$, $p = 0.7921$). (**e**) Percentages of detected bursts during intertrial intervals containing n spikes (Two-way ANOVA, opioid effect: $F(1,96) = 2.962$, $p = 0.0885$). Source data are provided as a Source Data file.

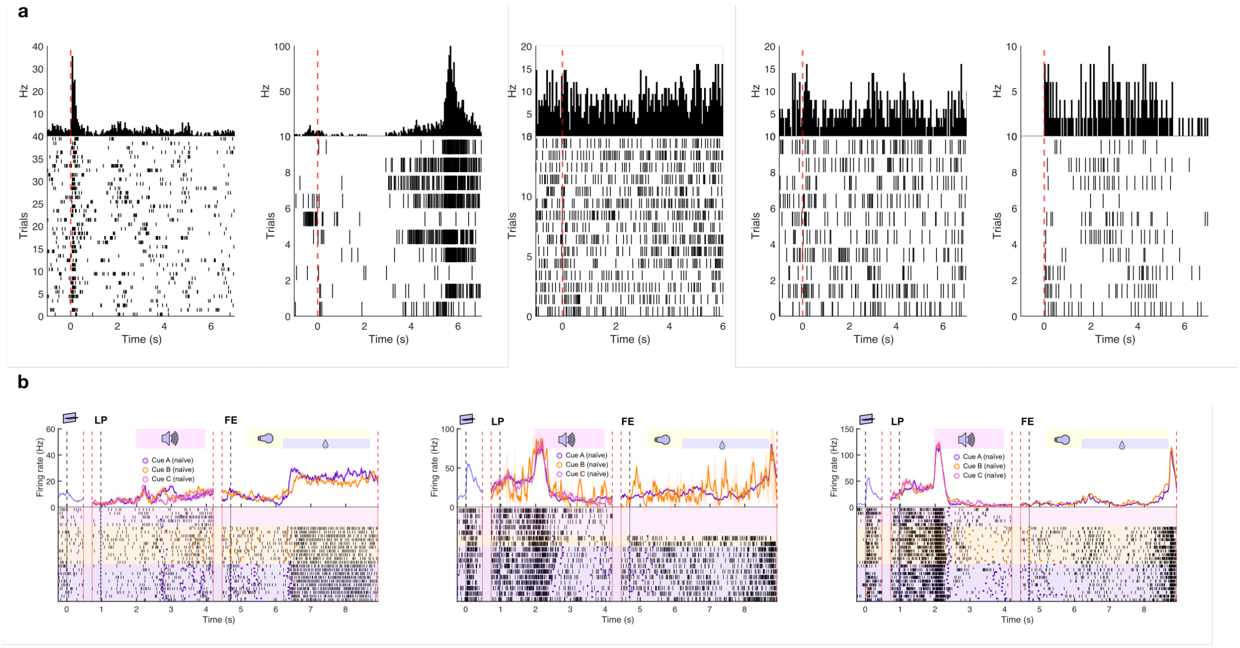

Supplementary Fig 9. Excluded units from Experiments 1 and 2.

(**a**)**,** PSTH and raster data from the five units manually removed from the putative dopamine cluster in Experiment 1. Activity centered around sucrose cue onset (0 s). The first unit on the upper left was removed to avoid duplication as it was simultaneously recorded in a nearby channel (Supplementary Fig. 2, i to l). The other units were removed due to concern of misclassification (lack of phasic cue response). (**b**), PSTH and raster data from the three units manually removed from the putative dopamine cluster in Experiment 2, displayed as in Fig. 5. The units were removed due to concern of misclassification (sustained non-phasic responses in unit 1 and extreme outliers in firing response to auditory cue in units 2 and 3).

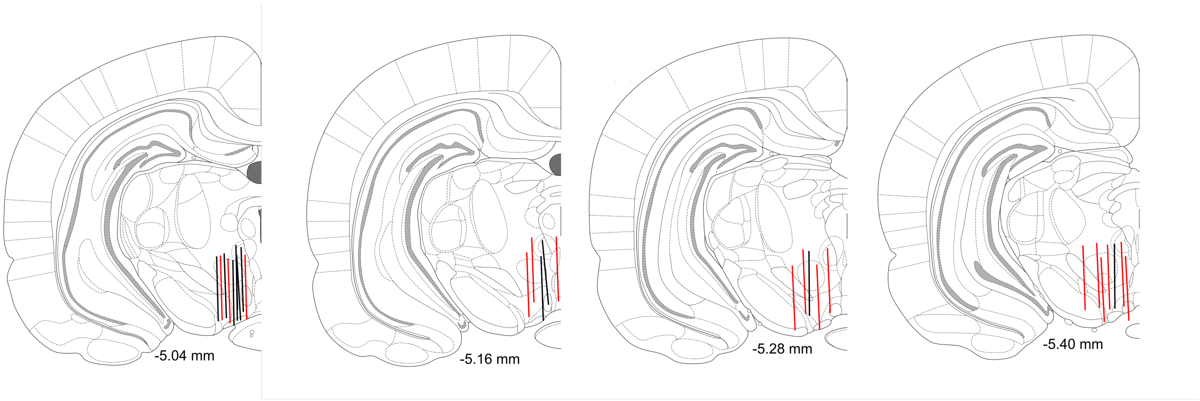

Supplementary Fig 10. Histological verification of electrode placement.

Lines show final placement of electrode track (N = 22 rats). Red lines correspond to electrodes in Experiment 1, and black lines correspond to Experiment 2. Numbers indicate antero-posterior position relative to Bregma.
